# Targeting the hallmarks of aging: mechanisms and therapeutic opportunities

**DOI:** 10.3389/fcvm.2025.1631578

**Published:** 2025-07-01

**Authors:** Fumihiro Sanada, Shinichiro Hayashi, Ryuichi Morishita

**Affiliations:** Department of Clinical Gene Therapy, Osaka University Graduate School of Medicine, Osaka, Japan

**Keywords:** chronological aging, biological aging, aging related disease, senscence, chronic inflammation

## Abstract

Aging is a complex biological process characterized by a gradual decline in cellular and physiological function, increasing vulnerability to chronic diseases and mortality. It involves a set of interconnected mechanisms known as the hallmarks of aging, including genomic instability, telomere attrition, epigenetic alterations, loss of proteostasis, mitochondrial dysfunction, cellular senescence, stem cell exhaustion, altered intercellular communication, and dysregulated nutrient sensing. These processes act at molecular, cellular, and systemic levels, contributing to age-related disorders such as neurodegeneration, cardiovascular disease, and metabolic syndromes. Emerging therapeutic strategies aim to delay or reverse aging by targeting specific hallmarks. These include senolytics to eliminate senescent cells, NAD^+^ boosters and mitophagy inducers to improve mitochondrial health, epigenetic reprogramming, and caloric restriction mimetics such as metformin and rapamycin to modulate nutrient-sensing pathways. Advances in regenerative medicine, gene editing, and organ cross-talk modulation are also contributing to the development of personalized, multi-targeted anti-aging therapies. Integration of omics technologies and biomarker research is expected to enhance our ability to monitor biological aging and optimize interventions for healthy longevity. This review highlights the current understanding of the hallmarks of aging and explores potential treatment strategies in light of our recent findings.

## 1 Introduction

**Figure 1:**
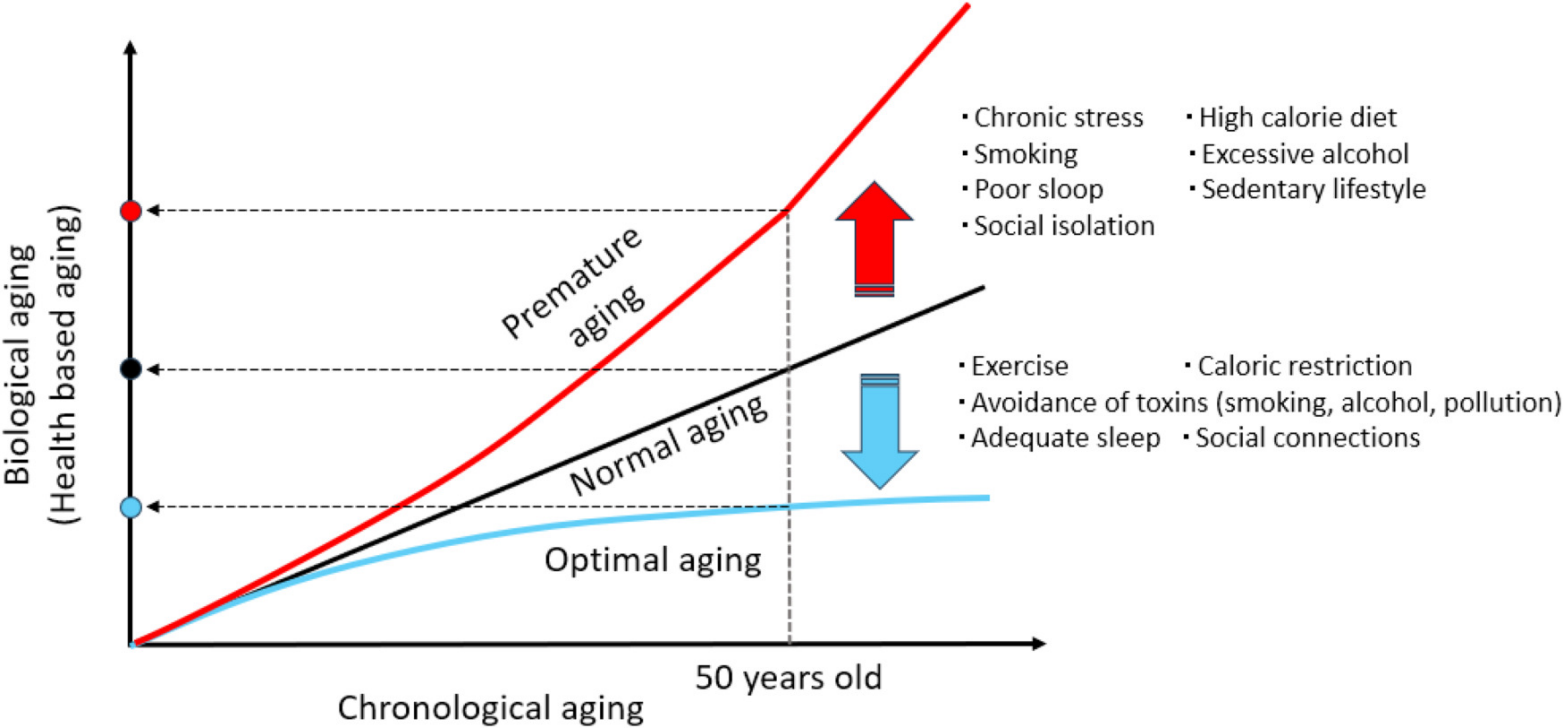
Chronological aging and biological aging. Three people are 50 years old chronologically, but one may be as healthy as a 40-year-old (optimal aging), another is age appropriate (normal aging), while another may have the biological characteristics of a 60-year-old (premature aging). Lifestyle has a profound influence on biological aging, either accelerating (premature aging) or decelerating (optimal aging) the process through its impact on cellular stress, inflammation, metabolism, and DNA stability, etc.

Aging is not merely the passage of time—it reflects how well the body maintains its function. Chronological age counts the number of years lived, while biological age (or health-based aging) reflects the actual condition of cells, tissues, and organs, revealing how quickly or slowly the body is aging (Figure 1). Biological age is shaped by the body's ability to maintain dynamic equilibrium—a stable internal environment despite ongoing internal and external stressors. This balance supports essential processes such as cellular repair, metabolic regulation, and immune function. When disrupted, it can lead to physiological decline, thereby accelerating aging and increasing susceptibility to age-related diseases.

This equilibrium is governed by a network of interconnected biological processes known as the “Hallmarks of Aging”—the key drivers of aging. First introduced in 2013, the hallmarks framework consolidated emerging scientific insights into the mechanisms of aging and identified potential points of intervention (1). In 2023, the hallmarks were updated to incorporate a decade of advances in both basic and clinical aging research (2). Each hallmark corresponds to a specific molecular or cellular alteration, often measurable through established biomarkers. These hallmarks progress in distinct yet interrelated stages, collectively contributing to aging phenotypes and age-associated diseases.

Ongoing research seeks to determine the optimal stages for therapeutic intervention and to identify lifestyle factors that may delay or prevent the onset of premature aging. In this review, we summarize the current understanding of the Hallmarks of Aging and present our related findings.

## 2 Hallmarks of aging and possible interventions

**Figure 2:**
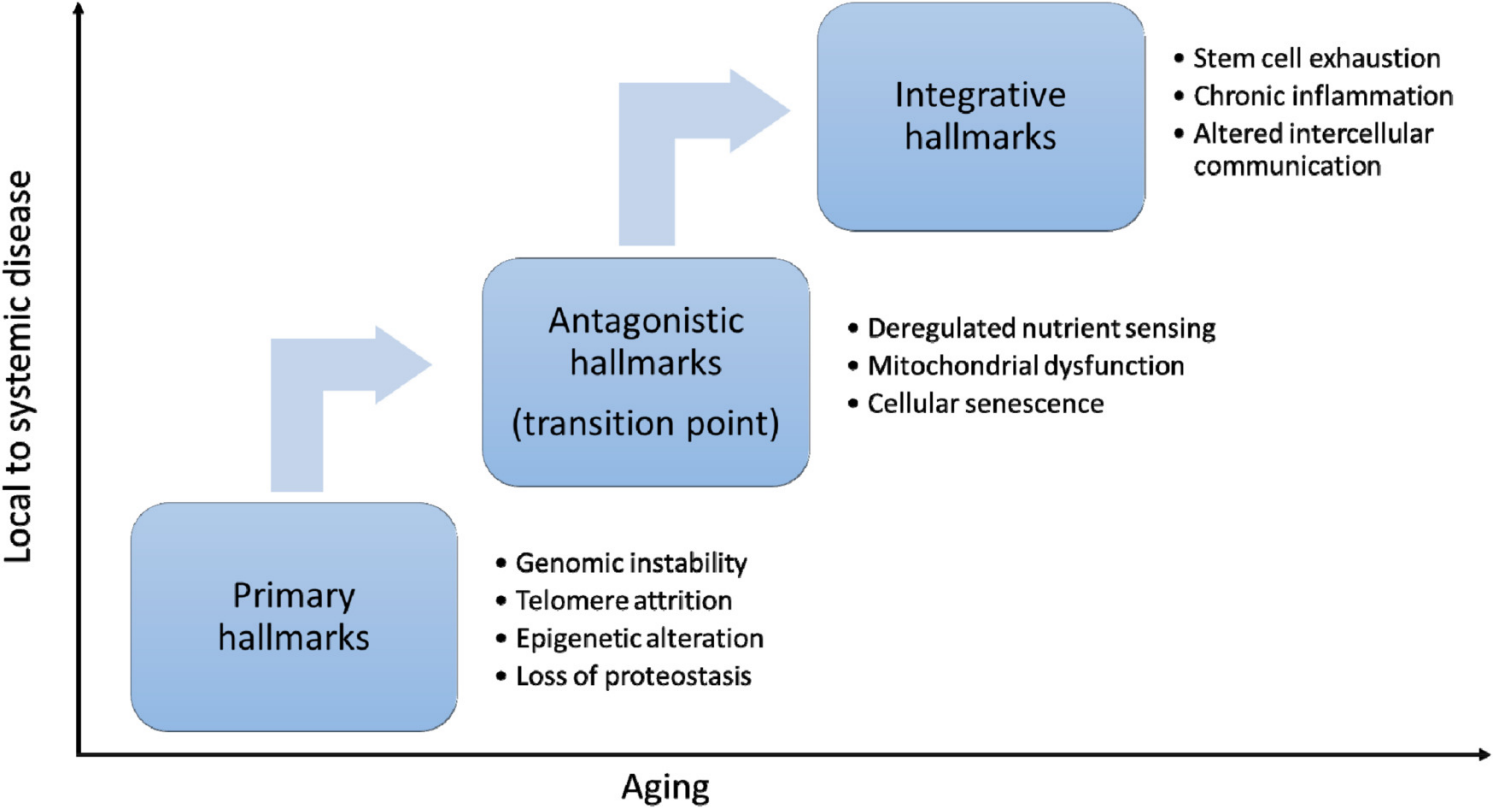
Hallmarks or aging. The hallmarks of aging can be categorized into three interconnected layers. Primary hallmarks—such as genomic instability, telomere attrition, epigenetic alterations, and loss of proteostasis—reflect the accumulation of molecular and cellular damage over time. In response, antagonistic hallmarks emerge as compensatory mechanisms, including deregulated nutrient sensing, mitochondrial dysfunction and cellular senescence. When these fail or become deleterious, they lead to integrative hallmarks, such as stem cell exhaustion, chronic inflammation, and altered intercellular communication, which drive systemic aging and functional decline. Antagonistic hallmarks represent a critical transition point in aging.

The hallmarks of aging are key biological processes that drive the progressive decline in function and increase the risk of age-related diseases (Figure 2). With recent advances in aging research, biological aging can now be modulated by targeting fundamental cellular and molecular processes (Table 1).

**Table 1:** Strategies for targeting the hallmarks of aging.

Hallmark	Category	Targeting strategy	Examples/Therapeutics
Genomic instability	Primary	Enhance DNA repair, reduce mutagenic stress	NAD^+^ boosters (NR/NMN), PARP activators, antioxidants
Telomere attrition	Primary	Promote telomerase activity, protect telomeres	TA-65, TERT gene therapy, lifestyle (stress reduction, exercise)
Epigenetic alterations	Primary	Modify chromatin state, reset epigenetic marks	Yamanaka factors, HDAC inhibitors, NAD^+^ precursors
Loss of proteostasis	Primary	Enhance protein folding and clearance	Rapamycin, spermidine, proteasome activators, HSP inducers
Deregulated nutrient sensing	Antagonistic	Restore metabolic balance via nutrient signaling pathways	Metformin, rapamycin, caloric restriction, intermittent fasting
Mitochondrial dysfunction	Antagonistic	Promote mitophagy, mitochondrial biogenesis	Urolithin A, MitoQ, exercise, NAD^+^ boosters
Cellular senescence	Antagonistic	Eliminate or suppress senescent cells and SASP	Senolytics (e.g., dasatinib, vaccine), metformin
Stem cell exhaustion	Integrative	Reactivate endogenous stem cells or replace with cell therapy	MSC therapy, GDF11, fasting, exercise
Altered intercellular communication	Integrative	Improve systemic signaling and tissue cross-talk	NAD^+^ boosters, probiotics, exercise, exosome therapy
Chronic inflammation (“Inflammaging”)	Integrative	Suppress inflammatory pathways and immune dysfunction	IL-1/IL-6 inhibitors, omega-3s, senolytics, anti-inflammatory diet

### 2.1 Primary hallmarks: the underlying causes of cellular decline

The primary hallmarks of aging—genomic instability, telomere attrition, epigenetic alterations, and loss of proteostasis—are considered the root causes of cellular aging. These hallmarks are associated with major age-related diseases: DNA damage accumulation contributes to cancer and Werner syndrome (3); telomere shortening is implicated in idiopathic pulmonary fibrosis and aplastic anemia (4); epigenetic dysregulation underlies Alzheimer's disease and Hutchinson-Gilford progeria syndrome (5); and impaired protein homeostasis is linked to neurodegenerative conditions such as Parkinson's and Huntington's diseases (6).

Interventions targeting these primary hallmarks—such as telomerase gene therapy to counter telomere attrition (7), NAD^+^ precursors like NMN to support DNA repair and genomic stability (8), partial epigenetic reprogramming with Yamanaka factors to reverse transcriptional aging (9), and autophagy enhancers like rapamycin to restore proteostasis (10)—have shown promise in preclinical models. However, translation to humans faces significant challenges, including cancer risk, off-target effects, delivery limitations, and insufficient long-term safety data. Therapies that stimulate cellular activity—such as those activating mTOR or telomerase—may paradoxically accelerate aging if not precisely regulated, leading to cellular senescence, stem cell exhaustion, or tumorigenesis (1, 7). Therefore, future strategies must emphasize precise, tissue-specific modulation, transient reprogramming, and combinatorial therapies that achieve rejuvenation without compromising long-term safety.

### 2.2 Antagonistic (secondary) hallmarks: protective responses that become detrimental

The antagonistic hallmarks of aging—deregulated nutrient sensing, mitochondrial dysfunction, and cellular senescence—initially serve protective or adaptive roles but become damaging when chronically activated. They are termed “antagonistic” because their effects vary depending on duration and context. Thus, these antagonistic hallmarks represent a critical transition point in aging.

Deregulated nutrient sensing, especially via the mTOR pathway, contributes to metabolic diseases such as type 2 diabetes and obesity through altered insulin/IGF-1 signaling (1). Mitochondrial dysfunction, characterized by impaired bioenergetics and oxidative stress, plays a central role in diseases like Alzheimer's, Parkinson's, and cardiomyopathy (11). Cellular senescence, marked by the secretion of inflammatory factors (SASP), contributes to conditions such as osteoporosis, osteoarthritis, pulmonary fibrosis, and cancer by promoting chronic inflammation and tissue dysfunction (12).

Modulating these hallmarks is a key goal of gerotherapeutics, with strategies including senolytics and caloric restriction mimetics. Recent research has explored vaccination as a novel approach. For example, a vaccine targeting CD153—a surface marker of senescent CD4^+^ T cells in visceral adipose tissue—was developed using a CD153 peptide conjugated with KLH and adjuvanted with CpG. It selectively reduced CD153^+^ senescent T cells via complement-dependent cytotoxicity, improving glucose tolerance and insulin sensitivity in obese mice (13). Another vaccination strategy targets GPNMB, a senescence-associated transmembrane protein enriched in vascular endothelial cells and leukocytes during atherosclerosis. Immunization against GPNMB reduced senescent cell burden, improved metabolic function, and alleviated atherosclerosis in high-fat diet-fed and ApoE-deficient mice. In progeroid mice, it ameliorated age-related phenotypes and extended lifespan, highlighting the therapeutic potential of targeting senescence-associated antigens (14).

While activating certain pathways may slow aging or treat diseases, chronic or unregulated stimulation can accelerate aging or promote cancer. For instance, we compared the effects of HGF and VEGF on endothelial progenitor cells under angiotensin II—a known atherosclerosis risk factor (15). HGF, but not VEGF, attenuated angiotensin II-induced senescence via suppression of the PI(3,4,5)P₃/Rac1 pathway and reduced oxidative stress. *In vivo*, HGF also enhanced neovascularization under angiotensin II stimulation, unlike VEGF. Although both factors promote angiogenesis in ischemic models, HGF has shown consistent clinical benefit in peripheral artery disease (PAD), whereas VEGF therapies have largely failed in Phase III trials. Notably, VEGF deficiency can induce aging-like features, but excessive VEGF contributes to pathological angiogenesis, inflammation, and cancer (16–19). Our data may explain these discrepancies (15). Therefore, temporal and tissue-specific control of modulators is essential to mitigate risks.

### 2.3 Integrative hallmarks: the downstream consequences of aging

As cellular damage accumulates and compensatory mechanisms fail, integrative hallmarks—stem cell exhaustion, chronic inflammation and altered intercellular communication—emerge. These reflect the systemic decline driven by earlier hallmarks and contribute to diseases such as sarcopenia and immunosenescence, where stem cell depletion impairs tissue regeneration (20), as well as chronic inflammatory conditions like atherosclerosis, Alzheimer's disease, and type 2 diabetes, exacerbated by SASP and systemic inflammaging (1, 2, 21).

We have reported that coagulation factor Xa induces cell senescence and activates inflammatory signaling through IGFBP-5, beyond its role in coagulation (22, 23). Similarly, Other groups also reported that low-grade activation of coagulation factor X contributes sterile chronic inflammation and atherogenesis via Protease-activated receptor-2 (24–28). Aging is associated with a hypercoagulable state involving increased procoagulant factors (e.g., fibrinogen, factors VIII and X, von Willebrand factor), endothelial dysfunction, reduced fibrinolysis, and increased platelet activity. This contributes to thrombotic events such as stroke and myocardial infarction, as well as microvascular damage, possibly linking coagulation and chronic inflammation in aging (29–31).

Therapies targeting integrative hallmarks include stem cell transplantation (32), heterochronic parabiosis and plasma exchange to rejuvenate systemic signaling (33), and senolytics like dasatinib and quercetin to reduce SASP (34). However, these approaches face limitations: immune rejection, short-lived effects, senescent cell heterogeneity, and delivery challenges.

Because integrative hallmarks involve multiple tissues and feedback loops, systemic targeting is inherently complex and prone to off-target effects. Early detection of localized aging (e.g., in the heart, kidney, or brain) is critical to prevent progression to systemic dysfunction. This can be achieved through biomarkers (e.g., NT-proBNP, creatinine, cystatin C), imaging (MRI, echocardiography), functional assessments (spirometry, gait speed), and emerging omics-based tools such as epigenetic clocks (35) and transcriptomic profiling (36).

## 3 Organs vulnerable to age-related damage and strategies for early detection

While aging affects all organs, those with high metabolic demands, limited regenerative capacity, and chronic exposure to stress—such as the brain, heart, kidneys, lungs, liver, bones, eyes, and skin—are particularly vulnerable. The brain is susceptible to neuronal loss, protein aggregation, and inflammation, increasing the risk of neurodegenerative diseases such as Alzheimer's (37). The heart undergoes arterial stiffening and myocardial hypertrophy, contributing to heart failure and arrhythmias (38). The kidneys experience nephron loss and vascular decline, elevating the risk of chronic kidney disease (39). Lung aging reduces tissue elasticity and impairs immune defense, making the lungs more prone to infections and chronic obstructive pulmonary disease (COPD) (40). The liver shows diminished detoxification capacity and accumulates fat, leading to non-alcoholic fatty liver disease (NAFLD) (41). Bone loss and cartilage degeneration result in osteoporosis and arthritis (42). The eyes are subject to oxidative stress, promoting cataracts and macular degeneration (43). Skin aging manifests as thinning, delayed wound healing, and UV-induced damage, which increases the risk of wrinkles and skin cancers.

A pivotal study by Dr. Tony Wyss-Coray's group at Stanford, published in *Nature* (44), demonstrated that organs can age at different rates—even in apparently healthy individuals. Using the SomaScan assay on blood samples from 5,676 participants, the team identified 856 organ-specific proteins across 11 organs and applied machine learning to estimate organ-specific biological age. The study revealed that: 18.4% of individuals over age 50 had at least one rapidly aging organ; 1.7% had multiple rapidly aging organs; Accelerated aging in 10 of 11 organs (excluding the intestine) was associated with a 15%–50% increased risk of mortality over 15 years. Notably, organ-specific accelerated aging was linked to: Heart: 250% higher risk of heart failure; Brain and vasculature: Strong predictor of Alzheimer's disease; Kidneys: Increased risk of hypertension and diabetes. These findings suggest that organ-specific biological aging could serve as an early warning system—potentially detectable through routine blood tests—even before clinical symptoms emerge. This implies that certain organs may undergo early biological aging (corresponding to primary and secondary hallmarks) and eventually engage in cross-organ communication that contributes to integrative hallmarks and systemic aging.

## 4 Conclusions and future directions

While aging is inevitable, its rate and impact can be modulated. A growing body of evidence suggests that specific behaviors and interventions can delay or attenuate the cellular and molecular hallmarks of aging, thereby enhancing both healthspan and lifespan (1). Lifestyle factors—including nutrition, physical activity, and stress management—play a critical role in regulating these hallmarks. On the other hand, several existing therapies—originally developed for other indications—are being repurposed or explored as anti-aging drug candidates. These drugs typically act by modulating key hallmarks of aging, such as genomic instability, cellular senescence, mitochondrial dysfunction, deregulated nutrient sensing, and stem cell exhaustion. Metformin, a widely used antidiabetic drug, has shown geroprotective potential by activating AMPK and inhibiting mTOR, thereby improving mitochondrial function and reducing inflammation and insulin resistance—key features of metabolic aging (45, 46). Rapamycin, an mTOR inhibitor originally used as an immunosuppressant, extends lifespan in multiple species by enhancing autophagy, improving proteostasis, and reducing stem cell exhaustion (10, 47). Senolytics such as dasatinib and quercetin selectively clear senescent cells, reducing the pro-inflammatory secretory phenotype (SASP) and restoring tissue homeostasis in aged organisms (34, 48). NAD^+^ precursors, including nicotinamide riboside (NR) and nicotinamide mononucleotide (NMN), replenish declining NAD^+^ levels in aging tissues, enhance sirtuin activity, and improve mitochondrial function and DNA repair (8, 49). Acarbose, an *α*-glucosidase inhibitor, improves glucose homeostasis and reduces postprandial insulin spikes, mimicking some effects of caloric restriction and extending lifespan in mice (50). Low-dose aspirin, through COX inhibition, exerts anti-inflammatory effects that may counteract chronic low-grade inflammation (“inflammaging”), although its net benefit in elderly populations remains controversial (51, 52). Lithium, used in bipolar disorder, inhibits GSK-3β and promotes autophagy and mitochondrial stability, with emerging evidence suggesting neuroprotective and potential lifespan-extending effects (53, 54).

SGLT2 inhibitors, such as empagliflozin, enhance metabolic flexibility, reduce oxidative stress, and may improve cardiovascular and renal aging phenotypes by mimicking fasting-like states (55, 56). The anti-aging effects of these existing drugs, the timing of administration, and long-term safety are expected to be studied. Looking ahead, it may become possible to detect organ-specific abnormalities before the emergence of integrative hallmarks and systemic dysfunction. Early identification of dysregulated nutrient sensing, mitochondrial dysfunction, or cellular senescence could enable timely, targeted interventions. Developing a simple, reliable method to monitor these early changes will be essential to identifying optimal therapeutic windows for age-delaying strategies.
